# Enhancing colorectal cancer prevention: a national assessment of public awareness in Egypt

**DOI:** 10.1186/s12889-024-18746-w

**Published:** 2024-05-27

**Authors:** Sania Ali Yehia, Mohamed Alboraie, Reham Ashour, Dalia Hassan, Reem Ezzat, Fathiya El-Raey, Mohammed Tag-Adeen, Mohamed Abdelaziz, Sabry Asfour, Abeer Abdellatef, Nermeen Abdeen, Rasha Elsayed, Sally Waheed Elkhadry

**Affiliations:** 1 Department of Epidemiology and Preventive Medicine, National Liver Institute, Menoufia University, Menoufia, Egypt; 2 Department of Internal Medicine Al-Azhar University, Cairo, Egypt; 3 Department of Hepatology and Gastroenterology, National Liver Institute, Menoufia University, Menoufia, Egypt; 4 Alexandria petroleum hospital, Alexandria, Egypt; 5 Internal Medicine Department, Assuit University, Assuit, Egypt; 6 Department of Hepatogastroenterology and Infectious Diseases, Damietta Faculty of Medicine, Al-Azhar University, Damietta, Egypt; 7 Department of Clinical Sciences, College of Medicine, Sulaiman Al Rajhi University, Al Qassim, Saudi Arabia; 8 Division of Gastroenterology and Hepatology, Department of Internal Medicine, Qena Faculty of Medicine, South Valley University, Qena Faculty of Medicine, Qena, Egypt; 9 Department of Clinical Oncology and Neuclear Medicine, Al Azhar University, Cairo, Egypt; 10 Department of Pediatrics, Matrouh General Hospital, Matruh, Egypt; 11 Department of Internal Medicine, Hepatogastroenetrology Division, Kasr Al-Aini Hospitals, Cairo University, Cairo, Egypt; 12 Department of Tropical Medicine, Faculty of Medicine, Alexandria University, Alexandria, Egypt; 13 Department of Internal Medicine Gastroenterology, Ain Shams University, Cairo, Egypt

**Keywords:** Public awareness, Prevention, Knowledge, Egypt, Risk factors, Colorectal cancer, Bowel cancer awareness measures

## Abstract

**Background:**

Despite the increasing incidence of colorectal cancer (CRC) in the Egyptian population, it still seems that there is a significant lack of awareness regarding the disease. This study aimed to assess the Egyptian population's awareness of CRC regarding its risk factors, the screening procedures, and the appropriate responses to its diagnosis.

**Method:**

A cross-sectional study was conducted in Egypt between July 2022 and March 2023 and recruited a convenient sample of adults from seven governorates representing different geographic areas, and socioeconomic and educational backgrounds with the help of the validated Bowel Cancer Awareness Measure (CAM) version 2.1. The modified Arabic questionnaire was validated through a pilot study including 30 patients. Then it was presented through a Google form before being shared via online methods and face-to-face interviews. The questionnaire provided both numerical and categorical data, which were analyzed accordingly. The Chi-square, the Fisher exact, and the Man-Whitney test were used to compare colorectal cancer poor and good knowledge groups. Logistic regression analysis was conducted to predict the factors that affected the awareness level of the study population.

**Results:**

Nine hundred forty individuals participated in the survey. Their ages ranged from 18 to 86 years old, with an average of 37.38 ± 12.22 years. The mean Knowledge score was 14.29 ± 7.05 out of 37 with most of our participants (71%) having poor knowledge about CRC. Most of the participants (64.1%) chose colonoscopy as the best screening modality, followed by an abdominal CT (27.8%), and fecal occult blood (15.5%). The study revealed significant differences between participants with good and poor knowledge of colorectal cancer. (78.5%) of participants with good CRC knowledge lived in cities, (85.4%) attained university or higher educational level, and (87.2%) of them were nonsmokers (*p* < 0.05%).

**Conclusion:**

In general, there was a lack of awareness about Colorectal cancer among the Egyptian population especially among rural and lower educational levels, and more health education campaigns are required to enhance CRC prevention efforts in Egypt.

**Supplementary Information:**

The online version contains supplementary material available at 10.1186/s12889-024-18746-w.

## Introduction

Colorectal cancer (CRC) is the third most common disease and second most fatal malignancy among both sexes combined worldwide. CRC has both evident environmental associations and genetic risk factors. Accumulation of genetic mutations either acquired or inherited in around ten to fifteen years is responsible for the change of the normal colonic epithelium to become a precancerous lesion and finally, an invasive carcinoma [1, 2].

The primary way of treating curable CRC is through surgery. While neoadjuvant chemotherapy isn't the best first line of therapy, it's becoming more common for locally advanced colon cancer before surgery. This method has been successful in reducing the size of the tumor and enhancing negative resection rates [3]. Adjuvant chemotherapy is primarily given subsequent to surgical intervention in patients striken with stage III and stage IV colon cancer [4]. Also, in rectal cancer in non-metastatic disease, preoperative (neoadjuvant) chemoradiotherapy or short course radiotherapy is recommended rather than initial resection followed by adjuvant therapy. While in metastatic unresectable disease, short course pelvic radiotherapy in combination with modern chemotherapy rather than chemotherapy alone is advised [5].

Immune checkpoint inhibitors (ICIs), chimeric antigen receptor (CAR) T cell therapy, T cell receptor (TCR) alterations, and cytokine therapy have recently came out as successful treatments for CRC. Also recent research on the use of probiotics [6], RNA-based therapies [small interfering RNA (siRNA), microRNA (miRNA), and RNA aptamer] [7], and oncolytic viral therapies [8] in the treatment of CRC have earning a promising results. In Egypt, colorectal cancer is considered a significant health problem, and its occurrence has been growing over the years. CRC is the 7th most common cancer in Egypt, accounting for 3.47% of male cancers and 3% of female cancers [9]. This rise is attributed to changes in diet habits and the aging population. According to studies, the rising CRC rates are correlated with increased alcohol intake, physical inactivity, high dietary fat, red meat, and processed foods; and low dietary fiber [10]. Smoking has been linked to colorectal adenomas as well as CRC incidence and mortality, which suggests that it may affect the prognosis of CRC patients as well [11].

Egyptians are diagnosed with CRC at later stages and have an overall survival of just two years [9], because there are no established practice national guidelines for CRC screening, and health insurance plans are insufficient. In Egypt, the national protocol is to screen only high-risk patients, e.g. a positive family history of CRC which has led to delayed symptomatic presentation [12]. Therefore, establishing an effective evidence-based screening program would help lay the groundwork for national guidelines and subsequent policy reforms.

Several major societies have developed various CRC screening methods depending on their availability. These include fecal occult blood test (FOBT), fecal immunochemical test (FIT), colonoscopy, flexible sigmoidoscopy, computed tomography (CT) colonography, and FIT DNA testing [13].

CRCs are easily prevented through CRC screening which can detect the disease during its early stages when the survival rates are high. To achieve this target, it is required to understand the levels of knowledge and awareness of the target population. This study was conducted to assess the awareness of the Egyptian population about colorectal cancer as regards risk factors of the disease, the screening process, and the initiative responses to its diagnosis. This would help in establishing an effective evidence-based screening program, laying the groundwork for national guidelines and subsequent policy reforms, enhancing CRC prevention efforts, and creating targeted health education campaigns to boost participation in screening.

## Materials and methods

Between July 2022 and March 2023, a cross-sectional study was conducted in Egypt to survey adults aged eighteen and above using a validated questionnaire. Egypt has twenty-seven governorates, four of which are urban and have no rural population (Cairo, Alexandria, Port Said, and Suez). The remaining twenty-three governorates are divided into urban and rural areas, with nine located in the Nile Delta and Nile Valley and the remaining five located on the country's eastern and western boundaries. The study was conducted in seven governorates representing urban, lower Egypt, Upper Egypt, and frontier governorates (Cairo, Alexandria, Menoufia, Damietta, Assiut, Quena, and Matrouh) The study aimed to include individuals from all educational and socioeconomic levels and not to dismiss rural and lower educational and socioeconomic backgrounds, and participants were recruited through face-to-face interviews conducted by trained interviewers from National Liver Institute, Menoufia University in Menoufia governorate. In other governorates, online methods such as Google Forms were used and distributed via email, WhatsApp, and other social media. The sample size was calculated using Epi-Info software to be 384 subjects with a 95% confidence interval, 80% power, an expected frequency of 50%, and a margin of error of 5%. Finally, a total of 940 participants were included in the study.

### Sampling method

The study participants were selected using convenience sampling from urban, Lower Egypt, Upper Egypt, and frontier governorates.

### Inclusion and exclusion criteria

Egyptians over the age of eighteen were eligible for the study, while those under eighteen and those who refused participation were excluded.

### Data collection and measurement tool

The public's level of awareness regarding colorectal cancer was assessed using the validated Bowel Cancer Awareness Measure (CAM) version 2.1, developed by University College London Cancer Research UK. The questionnaire was translated from English to Arabic by two bilingual healthcare workers with research and survey design experience. It was then back translated into English by another bilingual healthcare worker and modified to suit the cultural background of the study population. Additionally, five public health and gastroenterology professors reviewed the questionnaire for accuracy and content validity. A pilot study was conducted with thirty participants to assess questionnaire clarity, and the results were used to make adjustments to improve the tool. The data from the pilot study was not included in the final analysis. The tool Cronbach's alpha was 0.879, which is considered an acceptable level of internal reliability. The questionnaire has two parts. The first part collects information about the study participants' sociodemographic characteristics such as age, gender, residence (urban or rural), governorate of residence (urban, upper or lower Egypt and frontiers), educational level, occupation, marital status, special habits like smoking and alcohol intake, body weight, height, and history of having CRC either by the participant themselves or their partner (wife or husband), first degree relative, relative, friend, or anyone they know. The second part aims to assess participants' awareness of CRC and contains twelve main questions with a total of thirty-seven questions about the definition of colon and rectum, incidence of CRC, clinical picture (signs and symptoms), risk factors, screening and early detection, treatment, and prevention.

Participants were recruited using either in person by face-to-face interviews performed by trained interviewers on how to deal with different educational levels, especially low ones and to recruit participants and facilitate their completion of the study tool or online self-administered Google forms via various online methods (WhatsApp, mail, Facebook and other platforms) in Arabic language also face to face interview. Potential participants were contacted and informed about research objectives and invited to participate, furthermore, we asked participants to invite their contacts to participate in the study. The Checklist for Reporting Results of Internet E-Surveys (CHERRIES) [14] was followed to ensure the validity of the study results. A combined method of participant recruitment (in-person and online) was used to ensure a more diverse and representative sample of the study population and increase the generalizability of the study results.

### Ethical considerations

The study procedure was approved by the ethical committee of the National Liver Institute (NLI IRB 00003413 FWA0000227). The questionnaire used was anonymous and the confidentiality of the data was assured. All participants signed an Arabic informed consent form before enrolment in the study and had a full explanation of the study's aims and objective focusing on the point that their participation is completely voluntary.

### Statistical analysis

Data were collected and entered into the computer using the SPSS (Statistical Package for Social Science) program for statistical analysis, (BM Corp. Released 2013. IBM SPSS Statistics for Windows, Version 22.0. Armonk, NY: IBM Corp.).

The questionnaires provided both numerical and categorical data, which were analyzed accordingly. Quantitative data was presented as mean, standard deviation, and range, while qualitative data was presented as frequency and percentage. The Chi-square test was utilized to measure the relationship between qualitative variables, with the Fisher exact test being used for two-by-two qualitative variables when more than 25% of the cells had an expected count of less than 5. The Man-Whitney test was used to measure the association between quantitative variables when the data was not normally distributed. Logistic regression analysis was conducted to predict the factors that affected the awareness level of the study population. The *P*-value was considered statistically significant if it was less than 0.05.

Data were coded as the following correct answer was coded as (1) and incorrect answer was coded as (0) for the 5 points Likert scale questions, strongly agree or agree are considered as correct answers, and strongly disagree, disagree, and not sure as incorrect one. Total number of knowledge questions were twelve (q1 to q12). Some questions had underlining sub-questions as q5, q8, q9, q12. As follows; (Q5 had underlying 9 questions with 9 correct answers,Q8 & Q9 each had 5 correct answers to choose from, and Q12 had underlying 10 questions with 10 correct answers). CRC total awareness score ranged from (0–37) and study participants were divided into two awareness or knowledge score groups the first had poor knowledge (having a score of 50% or less score ranged from (0–18)) and the second was considered to have good knowledge (having score of > 50% score ranged from (19–37)).

## Results

The sociodemographic characteristics and special habits of the participants were analyzed in (Table 1). It showed that out of 940 participants who responded to the questionnaire; 58.2% were females while 48.8% were males. All participants were 18–86 years old with a mean age of 37.38 ± 12.22 years and a mean body mass index (BMI) of 28.6 + 5.39. The majority of the participants (44.8%) were from Lower Egypt, followed by 36.7% from urban governorates, 11.4% from Upper Egypt, and 7.1% from Frontier governorates. Most of the participants (72.1%) lived in urban areas, and 27.9% lived in rural areas. About one-third of the participants (33.1%) were employees, 26.7% were Health care workers (HCWs) and 27% did not have a job. About two-thirds of the participants (67.1%) were married and one-fourth (25%) were single. More than half of the participants (70.4%) have completed their university and above education level, 24% completed their secondary education level while illiterate people and those with basic education were 5.5%. A higher percentage of the participants (82.9%) were nonsmokers and only 6 participants consumed alcohol.

**Table 1:** Sociodemographic characteristics and special habits of the study participants (*N* = 940)

**Participants’ Criteria**		**N (%)**
**Age (years)**	**Mean** ± **SD**	37.39 ± 12.2
	**Median(min–max)**	36(18–86)
	**IQR**	28–44
**Gender**	**Female**	547 (58.2)
	**Male**	393 (41.8)
**BMI**	**Mean** ± **SD**	28.6 + 5.39
	**Median(min–max)**	27.97(16.62–61.69)
	**IQR**	24.88–31.52
**Governorates**	**Urban governorates**	345 (36.7)
	**Upper Egypt**	107 (11.4)
	**Lower Egypt**	421 (44.8)
	**Frontier governorates**	67 (7.1)
**Residence**	**Urban**	678 (72.1)
	**Rural**	262 (27.9)
**Occupation**	**HCW (Health care worker)**	251(26.7)
	**Employee and professional**	435(46.3)
	**Does not work**	254(27.0)
**Marital status**	**Single**	235 (25)
	**Married**	631 (67.1)
	**Widow**	44 (4.7)
	**Divorced**	30 (3.2)
**Education**	**Illiterate and basic**	52(5.5)
	**Secondary**	226(24.0)
	**University and above**	662(70.4)
**Smoking**	**Yes**	36 (3.8)
	**No**	779 (82.9)
	**Ex-smoker**	125 (13.3)
**Alcohol**	**No**	934 (99.4)
	**Yes**	6 (0.6)

For CRC-related history among the studied participants as shown in (Table 2), 95.4% never had CRC, while 1.2% of them had a history of CRC, there was a history of CRC in 1.1% of their partners, in 6.8% of their first-degree relatives, in 8.2% of their relatives, and in 7.8% of their friends, also 19% of them knew patients with CRC.

**Table 2:** Colorectal cancer-related history among study participants (*N* = 940)

**Variable**		**N (%)**
**Have you ever had colorectal cancer?**	Yes	11(1.2)
	No	897 (95.4)
	Not sure	30 (3.2)
	Prefer not to say	2 (0.2)
**Your partner (wife or husband) had colorectal cancer**	Yes	10 (1.1)
	No	891 (94.8)
	Not sure	37 (3.9)
	Prefer not to say	2 (0.2)
**Any of your 1st-degree relatives had colorectal cancer**	Yes	64 (6.8)
	No	814 (86.6)
	Not sure	57 (6.1)
	Prefer not to say	5 (0.5)
**Any of your relatives had colorectal cancer**	Yes	77(8.2)
	No	722 (76.8)
	Not sure	137 (14.6)
	Prefer not to say	4 (0.4)
**Your Friend had colorectal cancer**	Yes	73 (7.8)
	No	744 (79.1)
	Not sure	119 (12.7)
	Prefer not to say	4 (0.4)
**Does anyone you know have colorectal cancer?**	Yes	179 (19)
	No	607 (64.6)
	Not sure	147 (15.6)
	Prefer not to say	7(0.7)

### Colorectal cancer-related knowledge among study participants

Regarding CRC-related knowledge among our participants (Table 3), We revealed that the majority of them (73.1%) knew that the colon is the large intestine 67.1% knew that the rectum is the last part of the large intestine, nearly, one-third of participants knew that the functions of the colon are waste storage and water reabsorption, and only, 13.6% of participants knew that the incidence of colorectal cancer in Egypt is rare, and about two-thirds of them (62.9%) knew that there was a possibility of being cured from CRC.

**Table 3:** Colorectal cancer-related knowledge among study participants (*N* = 940)

Variable				N (%)	
1. Colon is	The large intestine*			687 (73.1)	
	The small intestine			38 (4.0)	
	The stomach			28 (3.0)	
	Stomach and small intestine			49 (5.2)	
	I don't know			138 (14.7)	
2. The rectum is	The last part of the stomach			53 (5.6)	
	The last part of the small intestine			38 (4.0)	
	The last part of the large intestine*			631 (67.1)	
	I don't know			218 (23.2)	
3. Colon function is	Food Digestion			143 (15.2)	
	Waste storage *			363 (38.6)	
	**Water reabsorption** *			253 (26.9)	
	Does not have a function			3 (0.3)	
	I don't know			178 (18.9%)	
4. The incidence of colorectal cancer in Egypt is	High			261 (27.8)	
	Average			551 (58.6)	
	Rare*			128 (13.6)	
5. Warning signs for colorectal cancer					
a) Do you think that rectal bleeding could be a sign of colorectal cancer?	**Yes** *			584 (62.1)	
	No			138 (14.7)	
	Don’t know			218 (23.2)	
b) Do you think that persistent pain in your abdomen (tummy) could be a sign of colorectal cancer?	Yes*			519 (55.2)	
	No			199 (21.2)	
	Don’t know			222 (23.6)	
c) Do you think that a change in bowel habits (diarrhea, constipation, or both) over weeks could be a sign of colorectal cancer?	**Yes** *			496 (52.8)	
	No			176 (18.7)	
	Don’t know			268 (28.5)	
d) Do you think that a feeling that your bowel does not empty after using the lavatory could be a sign of colorectal cancer?	**Yes** *			341 (36.3)	
	No			264 (28.1)	
	Don’t know			335 (35.6)	
e) Do you think that the presence of visible blood in your stools could be a sign of colorectal cancer?	**Yes** *			570 (60)	
	No			146 (15.5)	
	Don’t know			224 (23.8)	
f) Do you think that feeling pain in your back passage could be a sign of colorectal cancer?	**Yes** *			283 (30.1)	
	No			289 (30.7)	
	Don’t know			368 (39.1)	
g) Do you think that feeling a lump in your abdomen (tummy) could be a sign of colorectal cancer?	**Yes** *			537 (57.1)	
	No			117 (12.4)	
	Don’t know			286 (30.4)	
h) Do you think that tiredness/anemia could be a sign of colorectal cancer?	**Yes** *			368 (39.1)	
	No			230 (24.5)	
	Don’t know			342 (36.4)	
i) Do you think that unexplained weight loss could be a sign of colorectal cancer?	**Yes** *			483 (51.4)	
	No			155 (16.5)	
	Don’t know			302 (32.1)	
6. When do you screen for colorectal cancer?	At the onset of symptoms			657(69.9)	
	At the age of 20 years			88(9.4)	
	At the age of 50 years			173(18.4)	
	At the age of 70 years			22(2.3)	
7. Who is most likely to develop colorectal cancer?	A 20-year-old			9(1.0)	
	A 40-year-old			113(12.0)	
	A 60-year-old			194(20.6)	
	Bowel cancer is unrelated to age			624(66.4)	
8. What are the risk factors for colorectal cancer? (More than one option could be selected)					
Smoking	yes			60 (6.4)	
Inflammatory bowel disease	yes			99 (10.5)	
Family history of colorectal cancer	yes			109 (11.6)	
Fatty food	yes			180 (19.1)	
Colon polyps	yes			243 (25.9)	
don't know	yes			191 (20.3)	
9. What is the screening modality for colorectal cancer (more than one option could be selected)					
Fob(feacal occult blood)	yes			146 (15.5)	
Colonoscopy	yes			603(64.1)	
X-ray	yes			21(2.2)	
Ultrasound	yes			86 (9.1)	
CT scan	yes			261 (27.8)	
I don't know	yes			193 (20.5)	
10. Is it possible to be cured of colorectal cancer?	Yes			591 (62.9)	
	No			71 (7.6)	
	Don’t know			278 (29.6)	
11. Is there a relationship between colorectal cancer and irritable bowel syndrome?	Yes			259 (27.6)	
	No			229 (24.4)	
	Don’t know			452 (48.1)	
12. The following may or may not increase a person’s chance of developing colorectal cancer. How much do you agree that each of these can increase a person’s chance of developing colorectal cancer?	Strongly disagree N (%)	Disagree N (%)	Not sure N (%)	Agree N (%)	Strongly agree N (%)
a) Drinking more than 1 unit of alcohol a day	16 (1.7)	32 (3.4)	279 (29.7)	263 (28.0)	350 (37.2)
b) Eating less than 5 portions of fruit and vegetables a day	40 (4.3)	211(22.4)	417 (44.4)	200 (21.3)	72 (7.7)
c) Eating red or processed meat once a day or more	19 (2.0)	125 (13.3)	436 (46.4)	244 (26.0)	116 (12.3)
d) Having a diet low in fiber	22 (2.3)	115 (12.2)	425 (45.2)	264 (28.1)	114 (12.1)
e) Being overweight (BMI over 25)	13 (1.4)	126 (13.4)	437 (46.5)	237 (25.2)	127 (13.5)
f) Being over 70 years old	12 (1.3)	99 (10.5)	358 (38.1)	258 (27.4)	213 (22.7)
g) Having a close relative with colorectal cancer	8 (0.9)	66 (7.0)	255 (27.1)	322 (34.3)	289 (30.7)
h) Doing less than 30 min of moderate physical activity 5 times a week	31 (3.3)	191 (20.3)	426 (45.3)	205 (21.8)	87 (9.3)
i) Having a bowel disease (e.g. ulcerative colitis, Crohn’s disease)	5 (0.5)	43 (4.6)	315 (33.5)	353 (37.6)	224 (23.8)
j) Having diabetes	36 (3.8)	178 (18.9)	468 (49.8)	173 (18.4)	85 (9.0)
Knowledge score	Mean ± SD	14.29 ± 7.05			
	Median(min–max)	14 (0.00–32)			
	IQR	9.00—20.00			

Regarding the assessment of the awareness of warning signs for CRC, our results showed that most of our studied participants agreed that bleeding from back passage (62.1%), persistent abdominal pain (55.2%), change in bowel habits over weeks (55.8%), presence of blood in stools (60%), feeling an abdominal lump (57.1%) and unexplained weight loss (51.4%) could be signs for CRC. while nearly one-third of the participants thought that no complete bowel empty after using the lavatory, feeling pain in the back passage, tiredness, and anemia might be signs of CRC.

**Fig. 1:**
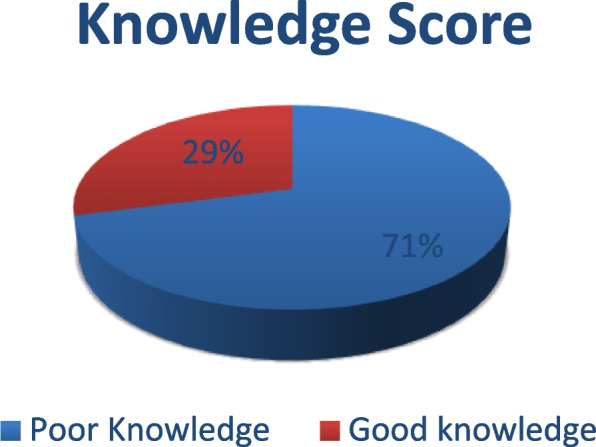
Distribution of CRC knowledge score groups among the participants (*N* = 940)

A large proportion of the participants (69.9%) thought that the optimal time of screening for CRC is at the onset of symptoms, 18.4% knew it should be at the age of 50 years while 2.3% thought that should occur at the age of 70 years. Most of them (66.4%) thought that the occurrence of CRC is unrelated to age and 25.9% of them knew that detection of colon polyp is a risk factor for CRC while 20.3% did not know any risk factor for CRC. Also, 27.6% of them thought there was a relationship between CRC and irritable bowel syndrome (IBS) while 48.1% of them did not know if there was a relationship between both diseases or not. The best screening modality for early detection of CRC was colonoscopy in most of the participants (64.1%), followed by an abdominal CT scan in 27.8% and fecal occult blood (FOB) in 15.5%, while 20.5% of them did not know any screening modality. By asking our participants about factors that may increase the person’s chance of developing CRC, our results showed that about one-third of them strongly agreed that drinking more than one unit of alcohol a day and having a close relative with CRC are considerable factors for developing CRC. 37.6% of them agreed that having a bowel disease (e.g. ulcerative colitis, Crohn’s disease) can increase the chance of CRC. Most of them were not sure about neither eating less than 5 portions of fruit and vegetables a day, eating red or processed meat once a day or more, eating low fiber diet, being overweight (BMI over 25), being older than 70 years, diabetic, nor doing less than 30 min of moderate physical activity 5 times a week, as factors which may increase the person’s chance for developing CRC. So the Mean Knowledge score was 14.29 ± 7.05 (Table 3), with most of our participants (71%) having poor knowledge about CRC (Fig. 1).

By comparing the socio-demographic characteristics, special habits, and colorectal cancer-related history between the two knowledge score groups of the study participants as shown in Table 4, we found that there were statistically significant differences between the two groups regarding their governorates, residence, occupation, educational level, smoking, and CRC related history in their relatives, friends and knowing people having CRC with (*P* value < 0.05%). in participants with good CRC knowledge: 78.5% of them were living in cities, 55.5% were HCWs, 85.4% reached university and above educational level and 87.2% were nonsmokers. Participants having positive CRC history in their relatives, friends, and anyone they knew (11.3%, 12.4%, 29.9%) respectively had a higher knowledge about CRC than those lacking this history (*p* < 0.05%).

**Table 4:** Comparison of study participants’ knowledge score groups regarding their sociodemographic characteristics, special habits, and CRC-related history

		**Knowledge**		***p*-value (chi-square test)**
		**poor (*N* = 666)**	**Good (*N* = 274)**	
**Age**	**Mean** ± **SD**	37.41 ± 12.301	37.35 ± 11.988	0.945*
	**Median(min–max)**	37(18–86)	35(18–78)	
	**IQR**	28–44	29–43	
**Gender**	**Male**	284(42.6)	109(39.8)	0.419
	**Female**	382(57.4)	165(60.2)	
**BMI**	**Mean** ± **SD**	28.75 ± 5.44	28.25 ± 5.18	0.28*
	**Median(min–max)**	28.01(16.62–61.69)	27.927(17.18–43.01)	
	**IQR**	24.91–31.99	24.83–30.72	
**Governorates**	**Urban**	270(40.5)	75(27.4)	**0.0001**
	**Upper**	58(8.7)	49(17.9)	
	**Lower**	308(46.2)	113(41.2)	
	**Frontier**	30(4.5)	37(13.5)	
**Residence**	**Urban**	463(69.5)	215(78.5)	**0.005**
	**Rural**	203(30.5)	59(21.5)	
**Occupation**	**Not work**	195(29.3)	59(21.5)	**0.0001**
	**Not HCW**	372(55.9)	63(23.0)	
	**HCW**	99(14.9)	152(55.5)	
**Marital status**	**Single**	165(24.8)	70(25.5)	0.637
	**Married**	444(66.7)	187(29.60)	
	**Widow**	33(5.0)	11(4.0)	
	**Divorced**	24(3.6)	6(2.2)	
**Education**	**Illiterate and basic**	49(7.4)	3(1.1)	**0.0001**
	**Secondary**	189(83.6)	37(13.5)	
	**University and above**	428(64.7)	234(85.4)	
**Smoking**	**Yes**	101(15.2)	24(8.8)	**0.032**
	**No**	540(81.1)	239(87.2)	
	**Ex-smoker**	25(3.8)	11(4.0)	
**Alcohol**	**No**	662(99.4)	272(99.3)	0.821* *
	**Yes**	4(0.6)	2(0.7)	
**Having Colorectal cancer**	**Yes**	4(0.6)	7(2.6)	0.06 * *
	**No**	639(95.9)	258(94.2)	
	**Prefer not to say**	1(0.2)	1(0.4)	
	**Don’t know**	22(3.3)	8(2.9)	
**Your partner (husband/ wife)** had colorectal cancer	**Yes**	5(0.8)	5(1.8)	0.08 **
	**No**	629(94.4)	262(95.6)	
	**Prefer not to say**	1(0.2)	1(0.4)	
	**Don’t know**	31(4.7)	6(2.2)	
**Your 1st-degree relative** had colorectal cancer	**Yes**	50(7.5)	14(5.1)	0.24
	**No**	567(85.1)	247(90.1)	
	**Prefer not to say**	4(0.6)	1(0.4)	
	**Don’t know**	45(6.8)	12(4.4)	
**Your relative** had colorectal cancer	**Yes**	46(6.9)	31(11.3)	**0.008**
	**No**	506(76.0)	216(78.8)	
	**Prefer not to say**	3(0.5)	1(0.4)	
	**Don’t know**	111(16.7)	26(9.5)	
**Your Friend** had colorectal cancer	**Yes**	39(5.9)	34(12.4)	**0.0001**
	**No**	525(78.8)	219(79.9)	
	**Prefer not to say**	4(0.6)	0(0)	
	**Don’t know**	98(14.7)	21(7.7)	
Anyone you know had colorectal cancer	**Yes**	97(14.6)	82(29.9)	**0.0001**
	**No**	443(66.5)	164(59.9)	
	**Prefer not to say**	6(0.9)	1(0.4)	
	**Don’t know**	120(14.6)	82(29.9)	

^*^Man -Whitney test

^*^^*^Fishers Exact test, Bold *p*-values indicating significance

In Multivariate analysis, the logistic regression model was statistically significant with a P value of less than 0.05. The model was able to explain 28.0% (Nagelkerke R2) of the variance in knowledge and accurately classified 77.9% of cases. The key CRC poor knowledge predictors as shown in (Table 5) included age, occupation, smoking, and educational level. There was a 2% decrease in poor CRC knowledge with an increase in age by a year [OR: 0.983, 95%CI: 0.97–0.996, *P*-value = 0.014].

**Table 5:** Predictors of CRC poor knowledge among study participants (*N* = 940)

		**B**	**Exp(B)**	**95% C.I for EXP(B)**		***P*-value**
				**Lower**	**Upper**	
**Age**		-.017	.983	.970	.996	0.014*
** Gender**	**Male (ref)**					
	**Female**	.374	1.454	.994	2.127	0.054
** Residence**	**Urban(ref)**					
	**Rural**	-.176	.839	.570	1.235	**0.373**
**Occupation**	**HCW (ref)**					0.0001*
	**Unemployed**	1.366	3.921	2.590	5.934	0.0001*
	**Not HCW**	2.305	10.023	6.695	15.005	0.0001*
**Education**	**University and above(ref)**					0.0001*
	**Basic and illiterate**	1.977	7.219	2.126	24.517	0.002*
	**Secondary**	.972	2.644	1.733	4.034	0.0001*
**Smoking**	**No (ref)**					0.102
	**Ex smoker**	.309	1.362	.580	3.196	0.478
	**Yes**	.626	1.870	1.046	3.345	0.035*
**Constant**		-.282	.754			0.382

B the unstandardized regression coefficient

Exp(B) the exponentiated coefficient, indicating the adjusted odds ratio

95% C.I. the 95% confidence interval, *and Bold p-values indicating significance

The odds of having poor CRC knowledge among study unemployed participants were about 3.921 times higher than the corresponding odds for HCW (Healthcare Workers) [OR: 3.921, 95%CI: 2.590–5.934, *P*-value = 0.0001]. The odds of having poor CRC knowledge among employee and professional participants were about 10.023 times higher than the corresponding odds for HCW [OR: 10.023, 95%CI: 6.695–15.005, *P*-value = 0.0001]. The odds of having poor CRC knowledge among participants with a basic and illiterate educational level were about 7.219 times higher than the corresponding odds for those of a university and above educational background [OR: 7.219, 95%CI: 2.126–24.517, *P*-value = 0.002]. Also, the odds of having poor CRC knowledge among secondary educated participants were about 2.644 times higher than the corresponding odds for those with a university and above educational level [OR: 2.644, 95%CI: 1.733–4.034, *P*-value = 0.0001].

## Discussion

This work investigates the degree of awareness regarding CRC among Egyptian people. To the best of our knowledge, this is the first study to investigate CRC awareness among Egyptians with different educational and Socio-demographic backgrounds. The study was carried out on 940 participants between the ages of 18—86 years old with different educational backgrounds to ensure good survey results from different groups. The majority of the participants (44.8%) were from Lower Egypt, followed by 36.7% from urban governorates, 11.4% from Upper Egypt and 7.1% from Frontier governorates. Most of the participants (72.1%) lived in urban areas, while 27.9% lived in rural areas.

Only 29% of our study participants had a good knowledge of colorectal cancer. This indicates a potential gap in public education about colorectal cancer and raises important considerations and the necessity of creating targeted and comprehensive awareness programs to improve understanding, early detection, and prevention of this type of cancer. This was in concordance with a Lebanese study which showed that 31% of study participants were aware of CRC [15]**,** Also, this was similar to studies carried out previously on the MENA (Middle East and North Africa) region that revealed poor knowledge of CRC [16, 17]. Al-Sharbatti et al. mentioned that more than half of the participants had poor knowledge considering CRC awareness in general [17], But this was different from Rocke K.D. which showed that two-thirds of the participants had good knowledge scores for CRC, mostly because the studied group was university students [18]. This demographic difference suggests that educational level and background significantly affects awareness and knowledge about CRC. University students have academic exposure and may have a higher baseline knowledge of health-related issues, including colorectal cancer. In contrast, our study represent a broader section of the population, with varying educational levels.

A large proportion of the participants (69.9%) suggested that the optimal time of screening for CRC is at the onset of symptoms. This belief indicates a lack of understanding of the preventive nature of screening programs. Additionally, exploring the reasons behind that misconception is of great importance. This may include misconceptions about the discomfort, invasiveness,, or potential risks associated with screening procedures may contribute to this belief. Healthcare providers may help by disseminating accurate information and addressing any misconceptions during patient interactions. In addition,Most of the participants (66.4%) thought that age has no correlation with the development of CRC. This misconception could potentially lead to false sense of security among younger age groups who may underestimate their susceptibility to the disease. This raises concerns about the need for providing accurate information via public health campaigns about the increased incidence of CRC with advancing age, public health campaigns can encourage individuals to make informed decisions about screening and adopt preventive measures in their lifestyle. and 25.9% of them knew that detection of colon polyp is a risk factor for CRC while 20.3% did not know any risk factor for CRC. The lack of knowledge regarding CRC risk factors, especially the connection between colon polyps and CRC, highlight the need for comprehensive and targeted educational interventions about colon polyps other known risk factors, such as age, family history, diet, and lifestyle choices. The knowledge of when to screen solely represents an obstacle against screening as mentioned by many studies [19, 20]. About 62% and 60% of the participants were aware of that back passage bleeding and fecal blood respectively may be signs of CRC. This was in concordance with the Lebanese study. that 68.2% of their study participants reported that 68.2% for anal bleeding and 65.3% for fecal blood, 39% for anemia, and 51.4% for unexplained weight loss as signs of CRC [15], this was mentioned in many other studies with close results in the Middle East and North Africa area and other countries besides another study carried out in Egypt [16, 19, 21–23]. 57% considered abdominal lump as a sign of CRC which is similar to a study that was carried out in Qatar [22] giving 56% to abdominal lump for diagnosing CRC. Unemptied bowel represented a risk factor for nearly a third of the participants and this was near to a study carried out by Power E et al. where 47% mentioned the same symptom as a risk factor [23] but opposite to the results gained by Huda T. Selim et al. which showed that only 9% thought un-empty bowel as a risk factor [20].

On analysis of the factors that may increase a person’s chance of developing CRC, our results showed that about one-third of participants agreed that drinking alcohol and positive family history of CRC are the most important factors for developing the disease. 37.6% defined bowel illness (e.g. ulcerative colitis, Crohn’s disease) as a factor that can increase the possibility of CRC. Diabetes, body mass index, and physical exercise were not much appreciated by the investigated groups, this was similar to other studies where physical exercise, diabetes, and dietary habits were at the bottom of the list as risk factors [24–27]. Most of the participants were not sure that dietary habits such as eating less than 5 portions of fruit and vegetables a day, eating red or processed meat once a day or more, and eating a low-fiber diet would be risk factors for developing CRC, this was different from the results seen in the study carried out by Huda T. Selim et al. [20]. And also opposite to some other studies that showed knowledge of dietary habits as more than half the participants showed strong agreement that diet is responsible for developing CRC [18, 27]. This disparity with previous studies highlights the difference in public understanding among different populations.

For the best screening modality for early detection of CRC, 64.1% of our study participants recommended colonoscopy as the best screening modality, followed by an abdominal CT scan in 27.8% and fecal occult blood (FOB) in 15.5%, this was opposite to De Bourcy et al. who reported FOB as the first choice for screening [28] and Tfaily MA et al. mentioned the same, as pain was an obstacle against using colonoscopy repeatedly for screening [15]. This difference may be attributed to various factors, including differences in healthcare systems, cultural backgrounds, and awareness levels. Furthermore, the screening preferences identified in our study underline the need for personalized approaches to encourage individuals to undergo CRC screening. Factors such as invasiveness, convenience, and perceived accuracy of the screening method can significantly influence individual preferences.

The educational level had a statistically significant impact in the degree of awareness in our studied group. This was in accordance with many other studies that reported positive correlation between higher educational levels and increased disease awareness [16, 21, 24, 29]. Also, awareness among health workers was more than other groups. This highlights the role of healthcare providers not only in delivering care but also in serving as valuable resources for disseminating health information to the general population. Regarding our participant`s Knowledge, it was significantly higher in study participants having positive CRC history in their relatives, friends, and anyone they knew (11.3%, 12.4%, 29.9%) respectively with (*P* value < 0.05%) than in negative ones. This is in concordance with another Egyptian study that was carried out among employees at Minia University [20]. This suggests that personal connections to individuals affected by CRC can serve as powerful motivators for individuals to search for and keep information about the disease. By understanding these demographic and background factors, public health interventions can be better designed to address the specific needs of various populations, eventually contributing to improved CRC awareness and prevention.

The main limitation of this study was depending on the convenience sampling method in recruiting its participants so sampling bias is unavoidable as most of our participants were recruited via the online method. however, efforts were made to increase the accuracy of results as The Checklist for Reporting Results of Internet E-Surveys (CHERRIES) [14] which was followed to ensure the validity of the study results. In addition, a combined method of participant recruitment (in-person and online) was used to ensure a more diverse and representative sample of the study population and increase the generalizability of the study results.

In summary, this is the first study to investigate the awareness of Egyptians towards colorectal cancer among participants taken from nearly all Egyptian governorates with variable sociodemographic and educational levels. The CRC awareness level, in general, was low among the Egyptian population, and more health education campaigns are required to enhance CRC prevention efforts in Egypt especially among rural and lower educational levels. Physicians should be encountered in these campaigns, particularly general practitioners, as they are the first line to pick up the cases of CRC.

### Supplementary Information

Supplementary Material 1.

Supplementary Material 2.

## Data Availability

No datasets were generated or analysed during the current study.
