# Supplementary material for: Enhancing colorectal cancer prevention: a national assessment of public awareness in Egypt
Source: BMC Public Health. 2024 May 27;24:1415. doi: 10.1186/s12889-024-18746-w (PMC11129470; doi:10.1186/s12889-024-18746-w)
Supplement: Supplementary file 1 — Supplementary Material 1. [file 12889_2024_18746_MOESM1_ESM.pdf]

Colorectal Cancer Awareness in Egypt

Could you tell me your age?

What is your gender? □**Male** □**Female**

What is your Occupation?

□**Not work** □**Worker** □**Employee**
□**professional** □**healthcare worker**

What is your marital status?

□**Single** □**Married** □**Widow**
□**Divorced**

What is the highest level of education qualification you have obtained?

□**Doctorate** □**Master** □**University**
□**Secondary**
□**Basic** □**Illiterate**

What is your governorate of residence?

Residence: □**Urban** □**Rural**

Weight ……………….Height……………..BMI……..………..

Special habits

Smoking: □**Yes** □ **No** □ **Ex-smoker**
Number of cigarettes ………duration……..

Alcohol intake: □ **Yes** □ **No**

Have you ever, had any family member or close friends or anyone you know have colorectal cancer?

	Yes	No	Not sure	Prefer not to say
a) You				
b) Partner (wife or husband)				
c) 1st-degree relatives				
d) Any of your relatives				
e) Close friend				
f) anyone you know				

1. **The Colon is:**
   - □**The large intestine**
   - □**The small intestine**
   - □**The stomach**
   - □**Stomach and small intestine**
   - □**I don't know**
2. **The rectum is:**
   - □**The last part of the stomach**
   - □**The last part of the small intestine**
   - □**The last part of the large intestine**
   - □**I don't know**
3. **Colon function is**
   - □**Digestion of food**
   - □**Waste storage**
   - □**Water reabsorption**
   - □**Does not have a function**
   - □**I don't know**
4. **The incidence of colorectal cancer in Egypt is**
   - □**High**
   - □**Average**
   - □**Rare**
5. **The following may or may not be warning signs for colorectal cancer.**

	Yes	No	Don't know
**a)** Do you think that rectal bleeding could be a sign of colorectal cancer?			
**b)** Do you think persistent pain in your abdomen (tummy) could be a sign of colorectal cancer?			
**c)** Do you think a change in bowel habits (diarrhea, constipation, or both) over weeks could be a sign of colorectal cancer?			
**d)** Do you think a feeling that your bowel does not empty after using the lavatory could be a sign of colorectal cancer?			
**e)** Do you think visible blood in your stools could be a sign of colorectal cancer?			
**f)** Do you think pain in your back passage could be a sign of colorectal cancer?			
**g)** Do you think a lump in your abdomen (tummy) could be a sign of colorectal cancer?			
**h)** Do you think that tiredness/anemia could be a sign of colorectal cancer?			
**i)** Do you think unexplained weight loss could be a sign of colorectal cancer?			

We are interested in your opinion

6. When do you screen for colorectal cancer?
   - □At the onset of symptoms
   - □At the age of 20 years
   - □At the age of 50 years
   - □At the age of 70 years

7. In the following years, who is most likely to develop colorectal cancer?
   - □A 20 year old
   - □A 40 year old
   - □A 60 year old
   - □ colorectal cancer is unrelated to age

8. What are the risk factors for colorectal cancer?
   - □Smoking
   - □Inflammatory bowel disease
   - □Family history of colorectal cancer
   - □Fatty food
   - □Colon polyps.
   - □I don't know

9. What is the screening modality for colorectal cancer?
   - □Fob (feacal occult blood)
   - □Colonoscopy
   - □X-ray
   - □Ultrasound
   - □CT scan
   - □I don't know

10. Is it possible to be cured of colorectal cancer?
   - □Yes
   - □No
   - □I don't know

11. Is there a relationship between colorectal cancer and irritable bowel syndrome?

   □Yes □No □I don't know

12. The following may or may not increase a person's chance of developing colorectal cancer. How much do you agree that each of these can increase a person's chance of developing colorectal cancer?

	Strongly disagree	Disagree	Not sure	Agree	Strongly agree
a) Drinking more than 1 unit of alcohol a day					
b) Eating less than 5 portions of fruit and vegetables a day					
c) Eating red or processed meat once a day or more					
d) Having a diet low in fiber					
e) Being overweight (BMI over 25)					
f) Being over 70 years old					
g) Having a close relative with colorectal cancer					
h) Doing less than 30 mins of moderate physical activity 5 times a week					
i) Having a bowel disease (e.g. ulcerative colitis, Crohn's disease)					
j) Having diabetes					

Thanks for your cooperation
