# Supplementary material for: Enhancing colorectal cancer prevention: a national assessment of public awareness in Egypt
Source: BMC Public Health. 2024 May 27;24:1415. doi: 10.1186/s12889-024-18746-w (PMC11129470; doi:10.1186/s12889-024-18746-w)
Supplement: Supplementary file 2 — Supplementary Material 2. [file 12889_2024_18746_MOESM2_ESM.pdf]

الوعي تجاه سرطان القولون والمستقيم بين أفراد المجتمع المصري

البيانات الشخصية والعائلية:

- **ما هو عمرك؟**
- **ما هو جنسك؟** □ذكر □ أنثى
- **ماهي مهنتك؟**
  □لا اعمل □عامل □موظف □متخصص □في القطاع الطبي
- **ما هى الحاله الاجتماعية؟** □ اعزب □متزوج □ارمل □مطلق
- **ماهو أعلى مستوى تعليمي حصلت عليه؟** □دكتوراه □ماجستير □جامعى
  □ثانويةعامه او فنى او ما يعادلها □ تعليم ابتدائي □ محوأميه □لايقرا ولا يكتب
- **مكان الإقامة؟** محافظة............ □ الحضر □ الريف
- **الوزن** .... **الطول** ....
- **التدخين :** □نعم ادخن حاليا □لا لم ادخن من قبل □كنت مدخن سابق
- **تناول الكحول :** □نعم □لا
- **هل أصبت لا قدر الله أنت أو أحد أفراد عائلتك أو أصدقاؤك المقربون بسرطان القولون والمستقيم ؟**

	نعم	لا	لست متأكد	افضل عدم القول
أ) أنت				
ب) زوجك /زوجتك				
ج) قريب من الدرجة الاولي من أفراد الأسرة				
د) قريب ليس من الدرجة الاولي من الاسرة				
هـ) صديق مقرب				
و) معارف				

1. **القولون هو:** □ الأمعاء الغليظة □الأمعاء الدقيقة
   □ المعدة □المعدة والأمعاء الدقيقة
   □لا أعلم
2. **المستقيم هو:** □آخر جزء من المعدة
   □الجزء الأخير من الأمعاء الدقيقة
   □الجزء الأخير من الأمعاء الغليظة
   □لا أعلم
3. **وظيفة القولون هي:** □هضم الطعام □تخزين بقايا الاكل
   □ امتصاص الماء □ليس لها وظيفة □لا أعلم
4. **هل نسبة الإصابة بسرطان القولون والمستقيم في مصر؟**
   □مرتفعة □ متوسطة □نادرة
5. **في رايك ؟ عما اذا كانت العلامات التالية تشير الي وجود سرطان القولون والمستقيم ام لا ؟**

	نعم	لا	لا أعرف
أ) هل تعتقد أن النزيف من الفتحة الشراجية يمكن أن يكون علامة على سرطان القولون والمستقيم ؟			
ب) هل تعتقد أن الألم المستمر في البطن يمكن أن يكون علامة على سرطان القولون والمستقيم ؟			
ج) هل تعتقد أن التغيير في الشكل المعتاد لعملية الاخراج (الإسهال أو الإمساك أو كليهما) على مدى أسابيع يمكن أن يكون علامة على الإصابة بسرطان القولون والمستقيم ؟			
د) هل تعتقد أن الشعور بعدم إفراغ أمعائك بعد استخدام المرحاض قد يكون علامة على الإصابة بسرطان القولون والمستقيم ؟			
ه) هل تعتقد أن الدم الظاهرفي البراز يمكن أن يكون علامة على سرطان القولون والمستقيم ؟			
و) هل تعتقد أن الألم في الممرالخلفي اسفل الظهر قد يكون علامة على الإصابة بسرطان القولون والمستقيم ؟			
ز) هل تعتقد أن وجود كتلة في البطن يمكن أن يكون علامة على سرطان القولون والمستقيم ؟			
ح) هل تعتقد أن الارهاق / فقر الدم يمكن أن يكون علامة على سرطان القولون والمستقيم ؟			
ط) هل تعتقد أن فقدان الوزن غير المبرر يمكن أن يكون علامة على الإصابة بسرطان القولون والمستقيم ؟			

6. **متى يتم عمل فحص للكشف عن سرطان القولون والمستقيم؟**
   □في بداية الأعراض □في سن ال 20 سنة
   □في سن الخمسين سنة □في سن السبعين.
7. **من هو الأكثر عرضة للإصابة بسرطان القولون والمستقيم ؟**
   □20 سنة □ 40سنة
   □ 60 سنة □ لا علاقة لسرطان القولون بالعمر
8. **ما هي العوامل التي تزيد من احتمال الاصابة بسرطان القولون والمستقيم ؟**

☐التدخين ☐مرض التهاب الأمعاءو القولون التقرحي
☐تاريخ عائلي للإصابة بسرطان القولون
☐الأطعمة الدهنية ☐زوائد لحمية بالقولون.
☐لا أعلم

9. ما هي طريقة الاكتشاف المبكر سرطان القولون والمستقيم ؟

☐تحليل لكشف دم خفي في البراز ☐ منظار القولون ☐الأشعة العادية
☐الموجات فوق الصوتية ☐الاشعة المقطعية ☐لا أعلم

10. هل يمكن الشفاء من سرطان القولون والمستقيم؟

☐نعم ☐لا ☐لا أعلم

11. هل هناك علاقة بين سرطان القولون والمستقيم ومتلازمة القولون العصبي؟

☐نعم ☐لا ☐لا أعلم

12. ما يلي قد يزيد أو لا يزيد من فرصة إصابة الشخص بسرطان القولون والمستقيم. إلى أي مدى توافق على أن اي من هذه العوامل يمكن أن يزيد من فرصة إصابة الشخص بسرطان القولون والمستقيم ؟

	اوافق بشدة	اوافق	غير متاكد محايد لا اعلم	اعترض	اعترض بشدة
أ) شرب أكثر من وحدة واحدة من الكحول في اليوم					
ب) تناول أقل من 5 حصص من الفاكهة والخضروات في اليوم					
ج) تناول اللحوم الحمراء أو المصنعة مرة واحدة في اليوم أو أكثر					
د) اتباع نظام غذائي منخفض الألياف					
هـ) زيادة الوزن (مؤشر كتلة الجسم أكبر من 25)					
و) تجاوز سن السبعين					
ز) وجود قريب من الدرجة اولي مصاب بسرطان القولون والمستقيم					
ح) ممارسة أقل من 30 دقيقة من النشاط البدني المعتدل 5 مرات في الأسبوع					
ط) الإصابة بأمراض القولون (مثل التهاب القولون التقرحي ومرض كرون)					
ي) الإصابة بمرض السكري					

(هذه المعلومات سرية ولن تستخدم الا لغرض البحث العلمي)

شكرا لحسن تعاونك معنا
